# Estimating long-term spatial distribution of *Plodia interpunctella* in various food facilities at Rajshahi Municipality, Bangladesh, through pheromone-baited traps

**DOI:** 10.1038/s41598-022-19036-4

**Published:** 2022-09-26

**Authors:** Md. Mahbub Hasan, Christos G. Athanassiou, Md. Akhtar Hossain

**Affiliations:** 1 Department of Zoology, Rajshahi University, Rajshahi, 6205 Bangladesh; 2 Laboratory of Entomology and Agricultural Zoology, Department of Agriculture, Crop Production and Rural Environment, University of Thessaly, Phytokou str., 38446 Nea Ionia, Magnesia Greece

**Keywords:** Entomology, Ecology, Ecological modelling

## Abstract

The Indian meal moth, *Plodia interpunctella* (Hübner), feeds on a wide range of commodities in most regions of the world. The present study presents six years of monitoring data for *P. interpunctella* in pheromone-baited traps by analyzing the trends of spatial variability, in five food facilities located in the Rajshahi municipality area of Bangladesh. We also tracked insect incidence at different spatial scales and evaluated the impact of food facility types and storage structures on insect populations. Our model showed an aggregated distribution pattern for *P. interpunctella.* Population patterns of *P. interpunctella* varied significantly among facilities. The highest number of moths captured was recorded in a grain retailer located at Municipal market, followed by a grain warehouse at Harian, pulse mill at Kazla, flour mill at Sapura and grocery shop at Katakhali. The population fluctuation of *P. interpunctella* moths was similar among the different locations tested, while there were no captures during the winter period. Our results indicate that long-term monitoring in a wide range of areas can be used to indicate population outbursts, under an area-wide management strategy.

## Introduction

Insect monitoring at the post-harvest stages of agricultural commodities is one of the key factors for practically implementing Integrated Pest Management (IPM). Monitoring of insects with traps is being considered as a standard approach in the IPM since early detection can help to minimize control operations through observing the status of pest population in the field^1–3^.Insect pest control in storage and processing facilities can be challenging since these facilities are spatially complex, dynamic, and features can vary considerably among locations^4^. The monitoring programs could be extending to appraise the effectiveness of prevention programs^5–9^. It also helps in determining the source of insects since insect migration can play a substantial role in recolonization following control applications, known widely as “population rebound”^10,11^. The monitoring program can be implemented to quantify areas where insect populations increase in number and may deserve further attention or treatment^4^. It is necessary to find out the most common approaches to be applied in food facilities for assessing insect activity since each facility has distinct spatial features that may seriously differentiate control measures. Larson et al.^12^ reported that the patterns of insect distribution enormously vary in food facilities as well as in food stock, suggesting that there are selective approaches in pest management practices at the spatio-temporal level. Several researchers investigated numerous monitoring studies on insect pest distribution in food facilities considering different factors including the number and location of traps, the structure as well as the economic cost^11,13–15^. They concluded that there were large dissimilarities in insect captures among different species^12^ and locations^10,15,16^ and emphasized the importance of targeted integrated pest management (IPM) plans based on specific conditions at the facility level^4,11,17,18^. Also, it has been demonstrated that a more complete scenario of the factors that influence the pest population patterns could be achieved while sampling over multiple years and under different environmental conditions that contribute to the spatial and environmental differences in insect captures^19^. In addition, the acquisition data will make information available on the reliability of insect spatial distribution and assist in developing IPM plans^1,20^.

The Indian meal moth, *Plodia interpunctella* (Hübner) (Lepidoptera: Pyralidae) is one of the major insect pests of a wide range of commodities in tropical and temperate regions^2^. Hamlin et al.^22^ studied comprehensively and categorized this species as a pest of grain, grain-based crops, and more than 20 different commodities, while Hagstrum and Subramanyam^23^ expanded this catalogue to more than 200 products.Although *P. interpunctella* is considered as a storage pest rather than as a field pest, it is particularly abundant outside of storage facilities, representing an important source of food infestation^10^. Soderstrom et al.^24^ and Campbell and Arbogast^11^ found high *P. interpunctella* activity outdoors and speculated that these moths were originating from outdoor sources, such as residues of the commodity. Several field studies revealed that the number of generations per year varied depending on different biotic and abiotic factors. It has been reported that there was only one to three generations of *P. interpunctella* completed in field in the USA and the high peak or peaks was usually recorded in the summer months^4,7,9,25,26^.

There are several methods which are currently being used for sampling of adult *P. interpunctella* populations in the field. The pheromone-based trapping of males has been considered as an important primary sampling tool of sampling^27–29^. The pheromone generally known as ‘‘ZETA’’ was one of the first commercial pheromones for stored-product insects, and has been used with success for monitoring of many stored product Pyralidae moths^26,30^. Nansen et al.^30^ mentioned that there are several factors that influence trapping, such as density, trap type, location, visual cues, pheromone composition and trap height. Hagstrum^31^ observed that the occurrence of *P. interpunctella* over a period of time differs between grain bins and locations and it is solely depended on the type of sampling method used. Campbell et al.^4^ determined the “hot-spots”, or places in which high numbers of *P. interpunctella* were sampled utilizing the contour mapping of pheromone-baited trap records, which are largely influenced by storing practices, presence of doors and windows, and other physical attributes of a facility^4^. However, most of the studies available on the utilization of pheromone-baited traps for monitoring of *P. interpunctella* are from facilities in Europe or North America, and are mostly focused on one or two years of monitoring. To our knowledge, there are no published reportson long-term monitoring of this species in food facilities in Bangladesh. At the same time, the vast majority of the monitoring data are focused on one single facility (e.g. a flour mill) and provide population fluctuation and distribution data for only a short period of time, and as such, the long-term spatial patterns in storage and processing facilities are still poorly understood. In this context, the present study was aims in determining the seasonal occurrence of *P. interpuncetella* at five food facilities located in Rajshahi municipality area in Bangladesh, through long-term monitoring (between 2014 and 2020). At the same time, apart from the seasonal occurrence of *P. interpunctella*, we also quantify the spatio-temporal distribution of this species at the different facilities, using in parallel different estimation indices.

## Results

**Figure 1:**
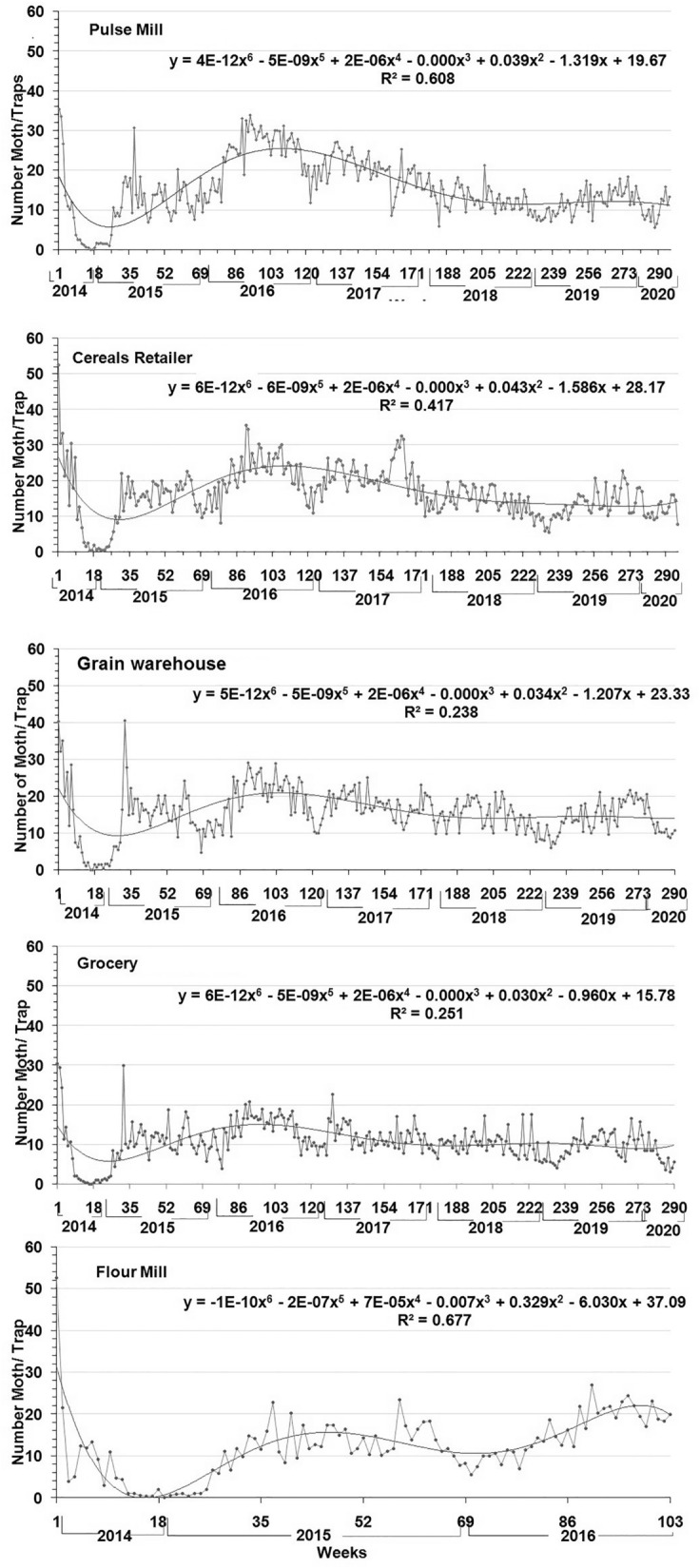
Mean number of *P. interpunctella*adultscaptured in pheromone traps at different food facilities in Rajshahi municipality area during the period from September 2014 to March 2020.

Population patterns of *P. interpunctella*, as these are depicted by pheromone-baited traps, varied significantly among the different treatments (F = 15.31; *df* = 301,5541; *P* < 0.001), and also among sites (F = 215.25; *df* = 4, 5541; *P* < 0.001), weeks (F = 13.39; *df* = 294,5541; *P* < 0.001) and trap sites (F = 6.83; *df* = 4,5541; *P* < 0.001)(Fig. 1). In the pulse mill at Kazla, the number of moths captured varied significantly (F = 5.31; *df* = 298, 1474; *P* < 0.001). Results also showed that there was significant variation among the weeks sampled (F = 5.37; *df* = 294,1474; *P* < 0.001).Similar trends were also noted at the grain retail facility at the Municipal market (months: F = 5.48; df = 298, 1474; *P* < 0.001;weeks: F = 5.50; *df* = 294, 1474; *P* < 0.001), the grain warehouse at Harian (months: F = 5.11; *df* = 298, 1474; *P* < 0.001; weeks: F = 5.16; *df* = 294, 1474; *P* < 0.001),the grocery shop at Katakhali (months: F = 4.46; *df* = 298, 1474; *P* < 0.001; weeks: F = 3.49; *df* = 294, 1474; *P* < 0.001) and the flour mill at Sapura (months: F = 6.52; *df* = 109, 420; *P* < 0.001; weeks: F = 6.05; *df* = 105, 420; *P* < 0.001).

**Figure 2:**
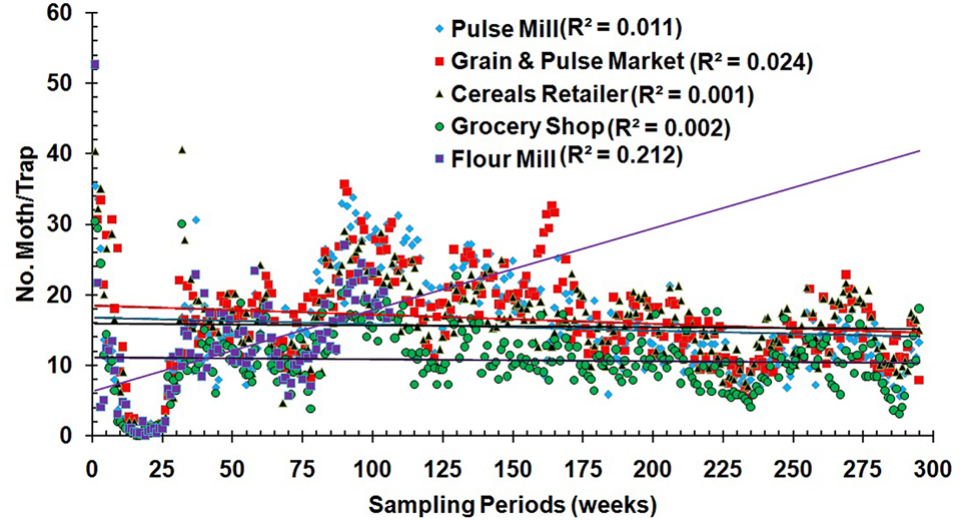
The random effect model for the population of *P. interpunctella* adults captured in pheromone-baited traps at different food facilities in Rajshahi municipality area during the period from September 2014 to March 2020.

The highest average number of moths was recorded in the grain retailer at the Municipal market, for the entire trapping period, followed by the grain warehouse at Harian, the pulse mill at Kazla, the flour mill at Sapura and the grocery stored at Katakhali (Table 1).The occurrence of *P. interpunctella* was increased from the beginning of trapping in September 2014, and after a peak on this period, was sharply decreased in the following interval towards the end of 2014 and early 2015 for all the sites studied. Moreover, there were no captures during winter months. An additional increase was noted in mid-2016, but after that period, captures were decreased again until early 2020 (Fig. 1).The population fluctuation of *P. interpunctella* can be depicted by sixth-degree polygonal lines (Fig. 1). R-square values indicate positive correlation between the sampling period and captures for all sites (range of r^2^ = 0.61–0.25). Nevertheless, the random-effects model assumes that the sampling period effects are uncorrelated with the population of *P. interpunctella* adults captured in the traps for all the food facilities (Fig. 2).

**Table 1:** Estimated parameters for spatial distribution of total population of Indian meal moth occurring in different food facilities at Rajshahi municipality area, Bangladesh during the five successive years (2014–2020).

Parameters	Sampling sites				
	Pulse mill	Grain retailers	Grain warehouse	Grocery shop	Flour mill
Mean	15.38	16.53	15.56	10.72	12.52
Range density = R	35.40	52.20	40.60	30.40	52.60
Variance (S^2^)	54.76	49.15	39.93	22.38	59.29
Standard Error (SE)	0.43	0.41	0.37	0.28	0.45
Median (Me)	14.00	16.20	15.80	10.40	11.90
Skewness (se: 14)	0.38	0.50	0.22	0.52	1.16
Kurtosis (se: 28)	− 0.18	2.09	1.45	2.14	5.36
Coefficient variance (CV)	48.11	42.41	40.62	44.12	61.50
Relative variation (RV)	2.80	2.47	2.36	2.57	3.58
Diffusion coefficient	3.56	2.97	2.57	2.09	4.74
Index of Lewis (I_L_)	1.89	1.72	1.60	1.44	2.18
Cassie index (Ca)	0.17	0.12	0.10	0.10	0.30
K value	6.01	8.38	9.93	9.86	3.35
Index of dispersion (I_D_)	1046.78	874.17	754.46	613.78	497.24
Z values	20.51	16.56	13.60	9.79	16.04
Index of mean clumping (I_DM_)	2.56	1.97	1.57	1.09	3.74
Lloyd’s mean crowding (X*)	17.94	18.50	17.13	11.81	16.26
Index of patchiness (I_P_)	1.17	1.12	1.10	1.10	1.30
Green’s index (GI)	0.01	0.01	0.01	0.00	0.04
Aggregation index (1/k)	0.17	0.12	0.10	0.10	0.30

**Figure 3:**
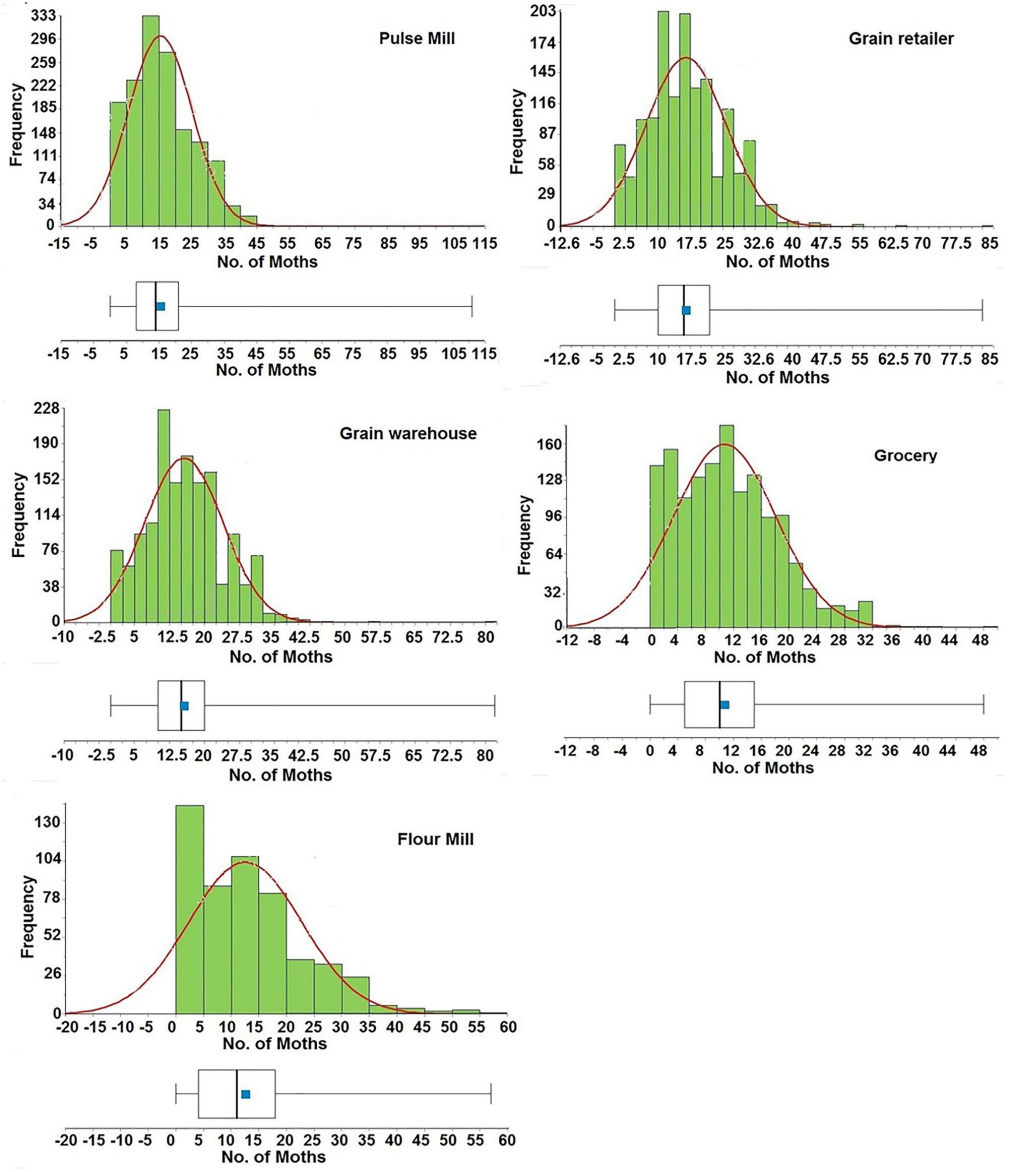
Histograms of the raw data for the weekly counts for *Plodiainterpunctella* adult captures in pheromone-baited traps at different food facilities located in Rajshahi municipality area.

The results of population distribution indicated aggregated patterns for all years (Table 1). Moreover, the distribution of data for all facilities was classified as normal since the values laid within the order of skewness and kurtosis (Table 1, Fig. 3).The index of Lewis for the entire population was significantly greater than one indicating a contagious dispersion (Table 1). In addition, the results of Cassie index (*Ca*) for the total population of the distribution was greater than zero which clearly indicated an aggregation distribution. On the contrary, the *K* values of the negative binomial distribution ranged between 3 and 9 for the six successive years, indicating random distribution. As spatial analyses showed, the index values of mean clumping (*IDM*) were positive for the negative binomial and Z-test values were greater than 1.96. The index of patchiness was greater than one and Green’s index (*GI*) was greater than zero, while its values were positive except for the grocery shop at Katakhali. All these indices clearly indicate an aggregated distribution for the entire trapping data for all six consecutive years. Moreover, the temporal changes in the spatial distribution pattern of *P. interpuncetella* population during the entire six-year period, showed that *1/k* (aggregation index)values were greater than zero. Lloyd's Index of Patchiness (*I*_*P*_) showed aggregated patterns, as values were greater than one (Table 1).

Correlation matrix analyses for the correlation coefficients show that there were strong associations in the number of moths captured in all facilities studied while using three test methods, i.e. Pearson's r, Spearman's rs and Kendall's tau (Table 2). Moreover, the highest correlation coefficient values were noted in relationship between the grain shop and grain retailer for all methods used, suggesting that these two facilities had a noticeable level of “synchronization” of captures (Table 2).

**Table 2:** Matrix Pearson’s correlation for the total population of Indian meal moth occurring in different food facilities at Rajshahi municipality area, Bangladesh during the five successive years (2014–2020).

Food facilities	Pulse mill	Grain shop	Grain retailers	Grocery shop	Flour mill
**Mill**					
Pulse	–	0.560	0.538	0.445	0.450
Grain shop	0.560	–	0.584	0.480	0.515
Grain retailers	0.538	0.584	–	0.565	0.518
Grocery shop	0.445	0.480	0.565	–	0.570
Flour mill	0.450	0.515	0.518	0.570	–

**Figure 4:**
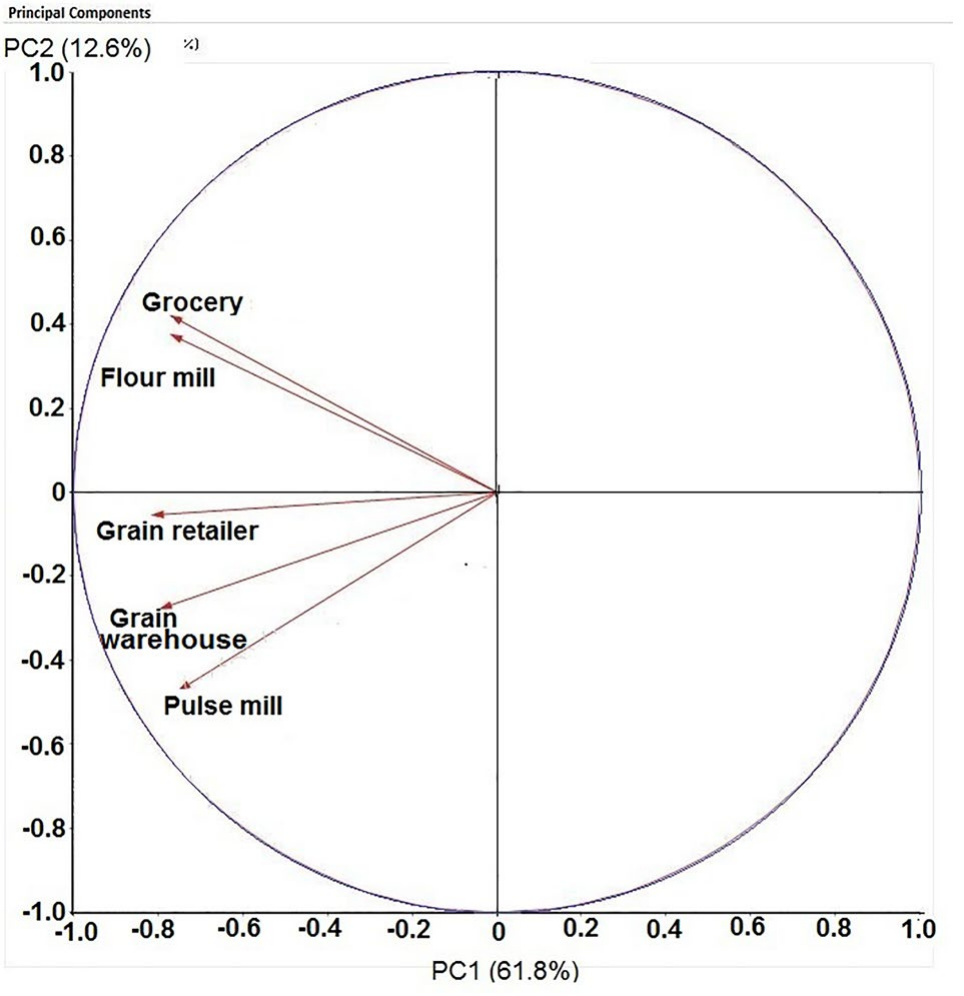
Principal Component Analyses for the weekly counts of *Plodia interpunctella* adult captures in pheromone-baited traps at different food facilities located in Rajshahi municipality area.

**Figure 5:**
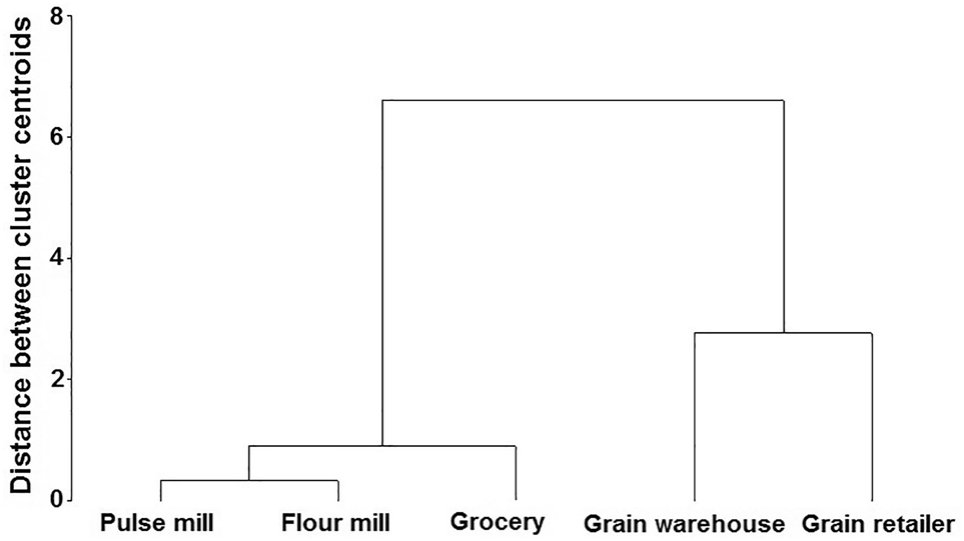
Hierarchical classification for the weekly counts of *Plodia interpunctella* adult captures in pheromone-baited traps at different food facilities located in Rajshahi municipality area.

The PCA plot exhibits that the grocery shop and flour mill are strongly correlated, as compared to the other sites (Fig. 4). In addition, the grain warehouse showed a weaker association with all the other facilities studied. In general, cluster analysis characterized two different main groups: a) a first main group that includes two subgroups, with the first subgroup formed by the pulse mill and the flour mill and the second subgroup included the grocery stored and b) a second main group including the grain warehouse and the grain retailer (Fig. 5).

## Discussion

The distribution and seasonal occurrence of *P. interpunctella* has been the subject of many studies in different types of facilities and commodities^2,21,23,32^. This species has an extremely wide variety of food preferences^23^, but it is generally regarded as a major pest of processed amylaceous commodities^21^. In our study we have found that there were differences in both seasonal occurrence and population density of *P. interpunctella* among the various facilities examined, but the overall data stand in accordance with the previous reports for the preference of the species to amylaceous products. Hence, although this species has been found to infest pulses and non-cereal based products, we generally found lower population densities in pulses, suggesting that pulses are not its preferred commodity. Conversely, the highest numbers were recorded for long intervals in the grocery shop and in the flour mill, which can be associated with the continuous presence of flour and related amylaceous products. Nevertheless, when these “preferred” commodities are not present, *P. interpunctella* can easily utilize “reservoir” products^4,11,17,21,32^. Although the seasonality in population fluctuation is somehow expected, periodical low population densities may create the false impression that the population densities are controlled, while in reality, this reduction is mostly related with temporally unfavorable abiotic conditions, such as temperature and humidity.

In an earlier study, Athanassiou et al.^32^ found that after the application of mating disruption in a storage and processing facility in Central Greece, the spatio-temporal distribution of adults of the Mediterranean flour moth, *Ephestia kuehniella* Zeller (Lepidoptera: Pyralidae) shifted to different areas, as a means of re-orientation and re-establishment in areas that were untreated or under dosed. A model for the distribution of *P. interpunctella* in South Korea underlined that importance of the external temperatures for the spread and establishment of this species indoors, and has shown that the dispersion is greatly enhanced by the vigorous flight activity of *P. interpunctella*, which can reach several kilometers^33^. As such, geographic information systems can be used with success to predict distribution and establishment of this species in areas that are currently considered as marginal for its development, and to time pest management measures^2,4,33,34^. In bulked grains, *P. interpunctella* larvae tend to remain in the same infestation patches throughout long periods of time^1,35^. In a food processing facility in USA, Campbell and Mullen^4^ found a considerable number of *P. interpunctella* adults that were captured in pheromone-baited traps that had been placed outside of the facility, but it was uncertain if these insects served as a “reservoir” population for the infestations inside the facility. In the case of the distribution patterns recorded here, association between different facilities may be due to their vicinity, and correlated well with the aggregated distribution of this species among the sampling units. This trend, however, is exhibited less vigorously in the case of some of the sites examined, which is considered as a consequence of the low population densities of this species for certain periods of time during the trapping period. Although expectable to some extent, these periodically low population densities may give the impression that the population of *P. interpuntella* is low, while it might be associated with spatial shifts in adjacent areas, as it is also evident from the “co-alteration” of the fluctuation among the different facilities. In this regard, trapping should be combined with sampling in the product, which can be further utilized to interpret trap data to actual infestation levels^35,36^, especially during the cold period of the year, where most of the individuals are expected to be at the larval or the pupal stage, and pheromone-baited traps may underestimate the actual population.

Considering the overall data, and despite seasonal variations, crowding and patchiness indices provide similar results for all facilities monitored, suggesting a strong aggregated distribution. Moreover, aggregation was more vigorous at facilities with high population densities of the species. This trend has been also recorded in the case of stored product beetle species, where the distribution was found to be aggregated, even when population densities were low^35–37^. For instance, in bulked grains stored in vertical silos Athanassiou and Buchelos^32^ reported that the most abundant beetle species had an aggregated distribution among sampling units, as it was shown by Taylor’s Power Law indices. Different indices, however, may provide different characterization of spatial pattern, affecting the accuracy of the sample plan and the optimum sample size^32,36^.

Inside temperature is apparently a critical factor for insect development and the fluctuation of population densities. However, it is not always possible to accurately monitor temperature levels in all facilities, and even if this is feasible, the results may be representative of specific indoor locations alone, and not to others. In this effort, utilizing historical data on the outside temperatures may be beneficial in estimating insect populations indoors and plan control strategies. The fluctuation of temperatures presented here indicates that captures of this species remained at high levels even when the outdoor temperatures were high. A viable paradigm in utilizing historical weather data is the case of grain aeration in silos, where the outside temperatures can predict the time for the initiation of aeration, and the need for subsequent fumigations^37^. Temperature data have been also found to be correlated well with the rebound of insect populations in storage and processing facilities^2,16,33,34^. In a similar way, the population rebounds can be predicted after the termination of the application of the killing agent, such as heat treatment, fumigation or fogging, where insects gradually recolonize the treated structures^10,16,38–40^. While we do not have quantitative data for the control measures that were taken in the facilities sampled, we estimate that spatial changes were also affected by these measures, and not solely by the abiotic conditions’ fluctuation or the presence of the commodity itself.

The current work presents a long-term monitoring data series for *P. interpunctella*, from a geographical area for which there are not that many data available so far. The study reveals that the populations in the different facilities had a noticeable correlation in their temporal dynamics, which indicates that these locations may be characterized as interconnected “demes” of a single meta population, rather than standalone independent populations. This should be seriously taken into account in designing control strategies, as there are areas where *P. interpunctella* populations could be treated “as a whole” and not through localized applications that just enhance a spatio-temporal shift.

## Methods

### Study sites

Monitoring of *P. interpunctella* was conducted for six years at weekly intervals, from 4 Sept 2014 to 19 March 2020 at different food facilities located in the municipality of Rajshahi area of Bangladesh where the products are temporally being stored before selling. The geographic coordinates of Rajshahi include as latitude: 24°22′26″N, longitude: 88°36′04″ and the elevation above sea level of approx. 23 m. There were five trial sites selected for sampling which include a pulse mill at Kazla area (site-1), a grain retailer at Municipal market (site- 2), a grain warehouse at Harian (site- 3), a grocery stored at Katakhali (site- 4) and a flour mill at Sapura industrial area (site- 5). The storage structure, storage management and practices in all the study sites were similar as these are built with concrete floors, brick walls and corrugated tin shed. The structural size (meter) of storage facilities were 12.19L × 9.14 W × 3.66H; 10.67L × 6.09 W × 3.05H;12.80L × 12.19 W × 4.57H; 7.62L × 9.14 W × 3.66H and 13.72L × 12.19 W × 4.57H for the sites- 1, -2, -3, -4, and -5 respectively. Moth monitoring was carried out from 4 September 2014 upto 8 September 2016 in the flour mill at Sapura area due to sudden shut down. The food products are being sold usually on a daily basis and stored for short periods in all sites.

#### Monitoring procedures

Diamond traps (Trécé, Adair OK, USA) were used for monitoring the indoor occurrence of *P. interpunctella*. These traps have a sticky surface to capture insects, baited with ZETA (Trécé, Adair OK, USA). In all food facilities, there were five traps per trial site and the traps were located in the same place throughout the entire monitored period.The number of moths captured were recorded weekly, while traps and lures were replaced as suggested by the manufacturer, except for the cases of excessively dusty areas or when traps were saturated with insect catches, at which time replacement was carried out more frequently. The traps were attached alongside the concrete wall at the height of 2 m from floor in all sites. Trapping locations were selected based on minimizing the practical factors suchas ease of servicing, reduced risk of damage, and location of attachment points.

#### Temperature and relative humidity

**Figure 6:**
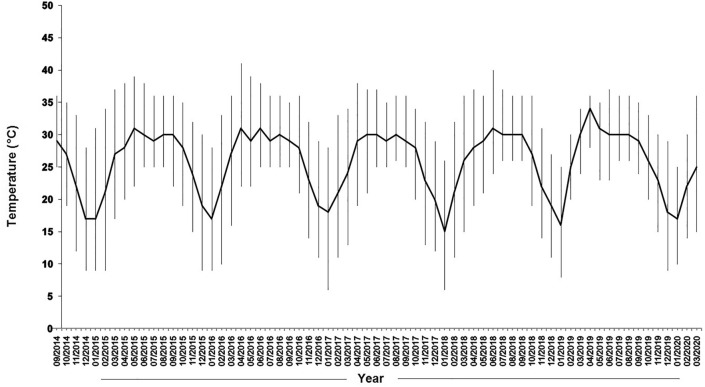
Temperature with the maximum and minimum recorded during surveying period atRajshahi, Bangladesh (Solid thick line indicates the average temperature in each month and thin line indicates maximum and minimum).

Temperature and relative humidity (r.h.) were estimated using data from Rajshahi Regional Weather Station. The distances of the sampling food facilities are 3.6, 6.8, 3.9, 4.7 and 9.4 km for site-1, site-2, site-3, site-4 and site-5 from the weather station respectively. These data were used to determine daily average with minimum and maximum temperatures (Fig. 6).

#### Statistical analysis

Assumptions of normality and homogeneity of variance were tested using Levene's^41^method and indicated that the data should be arcsine transformed before the analysis. Then, the data were analyzed through ANOVA using the PROC GLMMIX^42^, separately for each of the scenarios indicated above. The sample sites, weeks and position of the traps within each site were compared as fixed effects. Moreover, the correlated random-effects model was also fitted for making inferences on the population data based on the assumption of normal distribution of moths for all the sites. Principal Component Analysis (PCA) was performed to quantify the contribution of each factor during the storage period. Pearson’s correlation coefficients was used to measure the monotone association among the sampling locations.

### Spatial distribution pattern

The spatial distribution among the sample units was determined by eight indices of distribution and using two regression methods, these of Taylor^43^ and Iwao^44^. Such indices were chosen in an attempt to quantify dispersion patterns, based on specific aggregation indices. The comparable use of these methods in stored product sampling is given in detail by Subramanyam and Hagstrum^36^.

#### Distribution indices

Several estimates such as index of dispersion, clumping, crowdingand Green’s index were calculated^45^.

- Coefficient of variance (*C.V.*): To assess the fidelity of sampling for *P. interpunctella* population, the coefficient of variation values wereestimated as:
  $$C.V.=\frac{S}{X}\times 100$$
  where *S* is the standard deviation of the mean and *X* is the mean of population.
- Relative Variation (*R.V.*) is employed to compare the efficiency of various sampling methods^46^. The relative variation for the studied weeks was calculated as follows:
  $$R.V.=\frac{SE}{\overline{X} }\times 100$$
  where *SE* is the standard error of the mean and *X* is the mean of population.
- Index of dispersion (*ID*):
  $${I}_{DM}=\left(\frac{{S}^{2}}{\overline{X} }\right)- 1$$
  The index of dispersion is also known as the variance to mean ratio. Dispersion of a population can be classified through a calculation of the variance-to-mean ratio; namely:
- Diffusion coefficient:
  $\left(\frac{{S}^{2}}{\overline{X} }\right)$= 1 random distribution, < 1 regular distribution, and > 1 aggregated distribution (where, ${S}^{2}$ = sample variance;*X* = mean of population).
  Index of Lewis (*I*_*L*_):
- Lewis index was also calculated as per the formula given hereunder to determine the dispersion of *P. interpunctella.*
  $${I}_{L}= \sqrt{{S}^{2}/\overline{x} }$$
  The value of this index revealed > 1 contagious; < 1: regular and = 1 random distribution.
- Cassie index (*Ca*):
  $$Ca= \left(\frac{{S}^{2}}{\overline{X} }\right)/{\overline{X} }^{2}$$
  The spatial distribution pattern is aggregated, random and uniform when *Ca* > 0, *Ca* = 0 and *Ca* < 0, respectively^47^.
- The *K* value of negative binomial distribution:
  The parameter *k* of the negative binomial distribution is one measure of aggregation that can be used for insect species having clumped or aggregated spatial pattern. If*k* values are low and positive (*k* < 2), it indicates a highly aggregated population; *k* values ranging from 2 to 8 indicate moderate aggregation; and values higher than 8 (*k* > 8) indicate a random population^48^. The *k* values were calculated by the moment's method^49^, and given by:
  $$K={\overline{X} }^{2}/\left({S}^{2}-\overline{X }\right)$$
- Departure from a random distribution can be tested by calculating the index of dispersion (*I*_*D*_), where, n: denotes the number of samples:
  $${I}_{D}=(\mathrm{n}-1){s}^{2}/\overline{x }$$
  *I*_*D*_ is approximately distributed as *x*^*2*^with *n−*1 degrees of freedom. Values of *I*_*D*_which fall outside a confidence interval bounded with *n−*1 degrees of freedom and selected probability levels of 0.95 and 0.05, for instance, would indicate a significant departure from a random distribution.
  This index can be tested by *Z* value as follows:
  $$Z= \sqrt{2{I}_{D}}-\sqrt{2v-1}$$
  If 1.96 ≥ *Z* ≥ − 1.96, the spatial distribution would be random, but if *Z* < − 1.96 or *Z* > 1.96, it would be uniform andaggregated, respectively^50^ (Patil and Stiteler 1974).

*-* Index of mean clumping (*I*_*DM*_)^51^:

$${I}_{DM}=\frac{{S}^{2}}{X}- 1$$

The David and Moore index of clumping values increase with increasing aggregation. If the index value = 0, the distribution is random, positive value for negative binomial (aggregated) and negative value for positive binomial (regular).

- Lloyd’s mean crowding $$\left(\begin{array}{c}*\\ X\end{array}\right)$$:

Mean crowding $$\left(\begin{array}{c}*\\ X\end{array}\right)$$ was proposed by Lloyd to indicate the possible effect of mutual interference or competition among individuals. Theoretically, mean crowding is the mean number of other individuals per individual in the same quadrate:

$$\begin{array}{c}*\\ X\end{array}=\overline{X }+ \left[\left(\frac{{S}^{2}}{\overline{X} }\right)- 1\right]$$

As an index, mean crowding is highly dependent upon both the degree of clumping and population density. To remove the effect of changes in density, Lloyd introduced the index of patchiness, expressed as the ratio of mean crowding to the mean. As with the variance-to-mean ratio, the index of patchiness is dependent upon quadrate size^52^.

- Index of patchiness (*IP*): is dependent upon quadrate size.

$${I}_{P}= \left(\begin{array}{c}*\\ X\end{array} /\overline{X }\right)$$

If IP = 1 random, < 1 regular and > 1 aggregated.

*- Green’s index* (*GI*):

$$GI = \left[\left(\frac{{S}^{2}}{\overline{X} }\right)-1 \right]/(n-1)$$

This index is a modification of the index of cluster size that is independent of n^45^.

If GI > 0 or positive values are indicative of aggregation dispersion, GI < 0 or negative values indicative of uniformity or regular dispersion, and GI = 0 or negative values closer to 0 indicate randomness.

- To evaluate temporal changes in spatial pattern of *P. interpunctella*population during the studied years, an aggregation index (1/*k*)^53^was used.

It was calculated by the formula of

$$\frac{1}{k}= \left(\begin{array}{c}*\\ X\end{array} /\overline{x }\right)=1$$

where 1/k is aggregation index or Cassie’s index C and $$\left(\begin{array}{c}*\\ X\end{array} /\overline{x }\right)$$ is Lloyd’s patchiness index. The values of 1/*k* < 0, = 0, and > 0 represent regular, random and aggregated spatial pattern, respectively^54^.

### Ethical approval

In the frame of this study, no experiments have been conducted on animals or humans.

## Data Availability

The datasets generated during and/or analysed during the current study are available from the corresponding author on reasonable request.
